# Ablation of PPARγ in subcutaneous fat exacerbates age‐associated obesity and metabolic decline

**DOI:** 10.1111/acel.12721

**Published:** 2018-01-31

**Authors:** Lingyan Xu, Xinran Ma, Narendra Kumar Verma, Dongmei Wang, Oksana Gavrilova, Richard L. Proia, Toren Finkel, Elisabetta Mueller

**Affiliations:** ^1^ Genetics of Development and Disease Branch NIDDK National Institutes of Health Bethesda MD USA; ^2^ Shanghai Key Laboratory of Regulatory Biology Institute of Biomedical Sciences and School of Life Sciences East China Normal University Shanghai China; ^3^ Division of Endocrinology, Diabetes and Metabolism New York University New York NY USA; ^4^ Mouse Metabolism Core NIDDK National Institutes of Health Bethesda MD USA; ^5^ Center for Molecular Medicine NHLBI National Institutes of Health Bethesda MD USA

**Keywords:** aging, metabolic decline, obesity, PPARγ, subcutaneous fat

## Abstract

It is well established that aging is associated with metabolic dysfunction such as increased adiposity and impaired energy dissipation; however, the transcriptional mechanisms regulating energy balance during late life stages have not yet been fully elucidated. Here, we show that ablation of the nuclear receptor PPARγ specifically in inguinal fat tissue in aging mice is associated with increased fat tissue expansion and insulin resistance. These metabolic effects are accompanied by decreased thermogenesis, reduced levels of brown fat genes, and browning of subcutaneous adipose tissue. Comparative studies of the effects of PPARγ downregulation in young and mid‐aged mice demonstrate a preferential regulation of brown fat gene programs in inguinal fat in an age‐dependent manner. In conclusion, our study uncovers an essential role for PPARγ in maintaining energy expenditure during the aging process and suggests the possibility of targeting PPARγ to counteract age‐associated metabolic dysfunction.

## 1 INTRODUCTION

Adipose tissues are critical organs for energy homeostasis. While white fat (WAT) predominantly stores excess calories, brown adipose tissue (BAT) mostly contributes to energy utilization and thermogenesis (Farmer, 2008; Gesta, Tseng & Kahn, 2007; Mueller, 2014; Mueller, 2016). Recently, beige cells have been identified within subcutaneous white fat depots (iWAT): these cells display similar molecular and morphological characteristics to white adipocytes under basal conditions but acquire brown‐like features when exposed to low temperatures or β‐adrenergic stimuli and activate thermogenesis via the induction of uncoupling protein 1 (UCP1) and creatine metabolism (Kazak et al., 2015; Nedergaard & Cannon, 2014; Wu et al., 2012). Given the recent identification of both brown and beige fat cells in adult subjects in supraclavicular areas and the demonstration of their contribution to energy expenditure (Jespersen et al., 2013; Sharp et al., 2012; Wu et al., 2012), beige fat has become a potential target for the treatment of obesity and metabolic dysfunction.

In parallel with the increase in life expectancy observed over the last century (Christensen, Doblhammer, Rau & Vaupel, 2009), the incidence of obesity and metabolic dysfunction has also risen in the older population (Villareal, Apovian, Kushner & Klein, 2005). It has been shown recently that in addition to a decrease in BAT activity during the aging process, browning and beige fat cell function also decline late in life, potentially contributing to age‐associated metabolic dysfunction (Nedergaard, Bengtsson & Cannon, 2010; Rogers, Landa, Park & Smith, 2012). Thus, the identification of molecular mechanisms involved in the acquisition of brown‐like features in white fat during the aging process becomes an important step toward the development of treatments to counteract obesity and its metabolic consequences arising at late life stages.

The peroxisome proliferator‐activated receptor (PPAR) family of nuclear receptors is involved in the control of lipid and glucose homeostasis. PPARγ, one of the members of this subfamily, is required for the development of all types of fat cells and functions as a regulator of both white and brown gene programs in adipocytes (Lefterova, Haakonsson, Lazar & Mandrup, 2014; Tontonoz & Spiegelman, 2008). Ectopic expression of PPARγ in fibroblasts in vitro has been shown to drive adipogenesis (Mueller et al., 2002; Tontonoz, Hu & Spiegelman, 1994), and its selective ablation in fat leads to reduced adipose tissue mass and lipodystrophy (He et al., 2003; Jones et al., 2005; Wang, Mullican, DiSpirito, Peed & Lazar, 2013). In addition to the well‐documented role in coordinating gene expression programs of adipocyte differentiation and lipid storage, PPARγ has also been shown to directly bind to PPAR response element in promoters of brown fat‐selective genes, such as UCP1, Cidea, and Elovl3, and to induce them transcriptionally (Sears, MacGinnitie, Kovacs & Graves, 1996; Viswakarma et al., 2007; Kobayashi & Fujimori, 2012). Furthermore, its ligands, such as the antidiabetic drug rosiglitazone, have been shown to modulate brown remodeling of white adipose tissues in rodents and to increase energy expenditure, indicating a critical role of PPARγ in the browning process (Ohno, Shinoda, Spiegelman & Kajimura, 2012; Petrovic et al., 2009; Wilson‐Fritch et al., 2004). Genomewide binding studies have recently permitted the identification of PPARγ targets common to both white and brown fat tissues, as well as depot‐selective ones (Siersbæk et al., 2012). Given the unique capability of iWAT to adapt to different metabolic states by inducing either white or brown‐like gene programs, it is relevant to determine the contribution of PPARγ to the execution of each of these opposing functions in iWAT during the aging process.

Recent studies using genetic mouse models have revealed the importance of browning of iWAT in protecting from obesity and its metabolic consequences (Harms & Seale, 2013). In particular, it has been shown that adipose tissue ablation of regulators involved in thermogenic programs, such as PRDM16 or PGC1α, affects iWAT function and leads to diet‐induced obesity and metabolic alterations (Cohen et al., 2014; Kleiner et al., 2012). However, the analysis of the specific function of these regulators selectively in subcutaneous fat in vivo has been hindered by the lack of genetic methods to achieve iWAT‐specific deletion. Previously reported animal models carrying PPARγ ablation in every fat tissue generated by crossing PPARγ‐LoxP mice with either aP2‐ or adiponectin‐Cre mice revealed impaired fat development and reduced fat mass (He et al., 2003; Jones et al., 2005; Wang et al., 2013). Given that none of the existing Cre‐LoxP systems can permit the ablation of PPARγ selectively in subcutaneous fat during late stages of life, it remains to be determined whether PPARγ can specifically affect browning of subcutaneous tissue during the aging process.

Here, we achieved specific ablation or downregulation of PPARγ in inguinal adipose tissue via injections of adenoviruses expressing Cre or shPPARγ directly into iWAT of PPARγ‐LoxP or C57BL/6J aging mice, respectively, to assess the selective role of PPARγ in subcutaneous fat tissue at late life stages. Our studies show that aging mice with deficiency of PPARγ specifically in iWAT have increased adiposity, reduced energy expenditure, and insulin resistance. These effects are accompanied by decreased brown, but not white, fat gene programs, in iWAT. Direct comparison of the effects of PPARγ ablation in subcutaneous fat tissue of young and old mice revealed PPARγ preferential regulation of brown fat gene programs depending on the age of the mice. Collectively, these studies reveal an unexpected phenotype of mice with PPARγ deficiency during aging via activation of selective transcriptional programs during late life stages.

## 2 RESULTS

### 2.1 Ablation or downregulation of PPARγ selectively in subcutaneous fat tissue of mid‐aged mice via adenoviral injections

**Figure 1:**
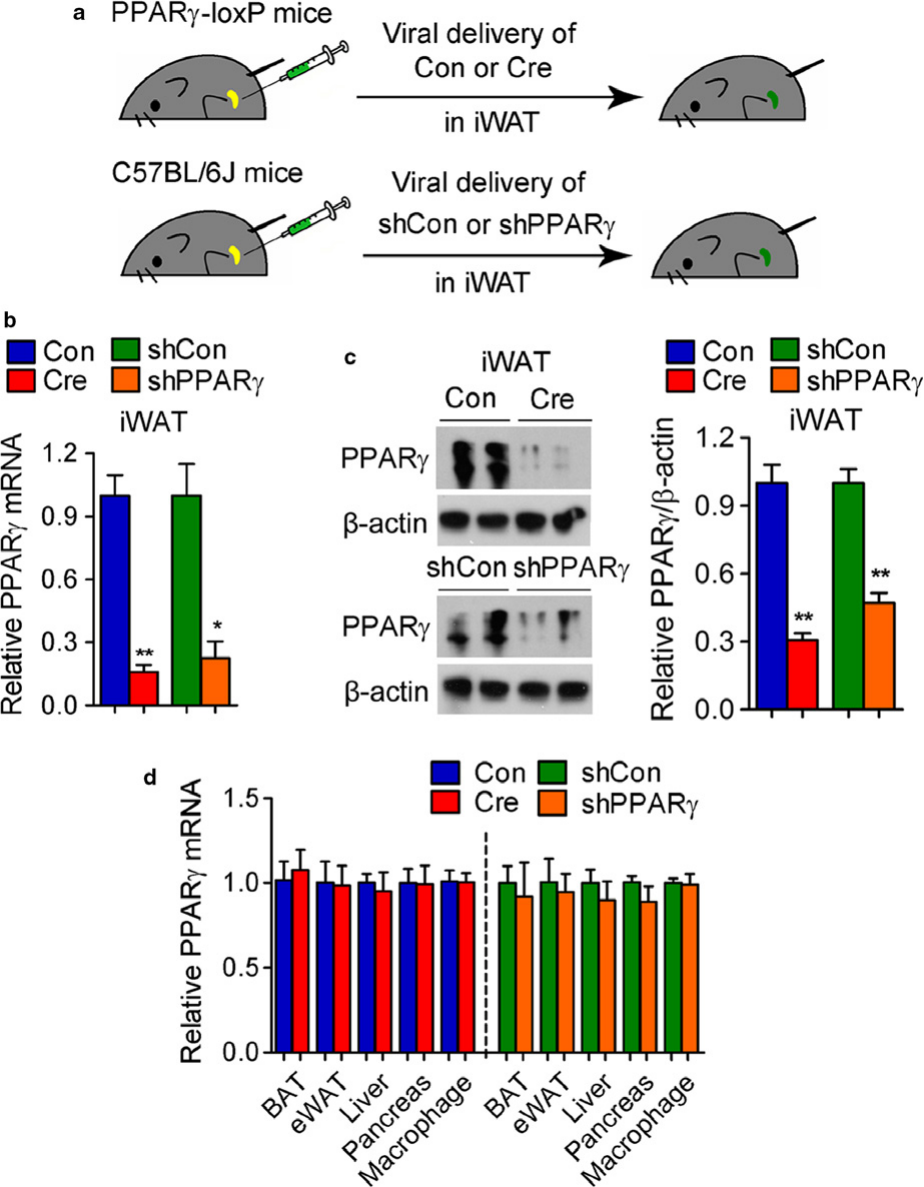
Selective ablation of PPARγ in iWAT of aging mice via adenoviral delivery. (a–d) Analysis of 12‐month‐old control, PPARγ‐iWAT‐KO, and PPARγ‐iWAT‐KD mice. *n *= 6 per group. (a) Illustration of the strategy used to achieve modulation of PPARγ levels in iWAT via adenoviral delivery. (b) mRNA and (c) protein levels of PPARγ in iWAT. (d) mRNA levels of PPARγ in fat depots, liver, pancreas, and macrophages present in iWAT. Data are presented as mean ± SEM and *, *p* < .05; **, *p* < .01

The requirements of PPARγ in adipose tissues have been extensively studied through the generation of mice with deletion of PPARγ in every adipose depot by crossing PPARγ‐LoxP with aP2‐ and adiponectin‐Cre mice; however, no reports have yet characterized the function of PPARγ specifically in iWAT in aged mice, in part due to the absence of genetic means to permit depot‐specific deletion of PPARγ at late life stages. To investigate the role of PPARγ specifically in iWAT during aging, we injected either control or Cre adenovirus into subcutaneous tissues of 12‐month‐old PPARγ‐LoxP mice. In addition, to achieve knockdown of PPARγ, we injected control or PPARγ shRNA adenovirus into 12‐month‐old C57BL/6J mice (Figure 1a). As shown in Figure 1b,c, injection of either Cre adenovirus in PPARγ‐loxP mice (PPARγ‐iWAT‐KO) or shPPARγ adenovirus in C57 mice (PPARγ‐iWAT‐KD) led to modulation of PPARγ levels in subcutaneous fat tissue. Importantly, adenoviral delivery did not affect PPARγ mRNA levels in epididymal WAT and BAT depots nor in tissues such as liver and pancreas (Figure 1d), suggesting the PPARγ deficiency was indeed achieved selectively in subcutaneous fat tissue.

Interestingly, we found no statistical difference in the levels of PPARγ mRNA in the total population of macrophages present in iWAT after injection of sh control or shPPARγ adenovirus (Figure 1d). To assess the differential efficiency in transduction of adenovirus in adipocytes and macrophages, we performed immunostaining using GFP and F4/80 antibodies in Ad‐GFP‐transduced iWAT and analyzed macrophages with single or double staining by confocal imaging (Fig. S1). The results of this analysis demonstrate that while adipocytes are transduced at a high percentage by GFP adenovirus (almost 100%), only a fraction (35.6 ± 14.7%) of macrophages present in fat tissue are also infected with GFP.

### 2.2 PPARγ ablation or downregulation in iWAT is associated with increased body weight and adiposity

**Figure 2:**
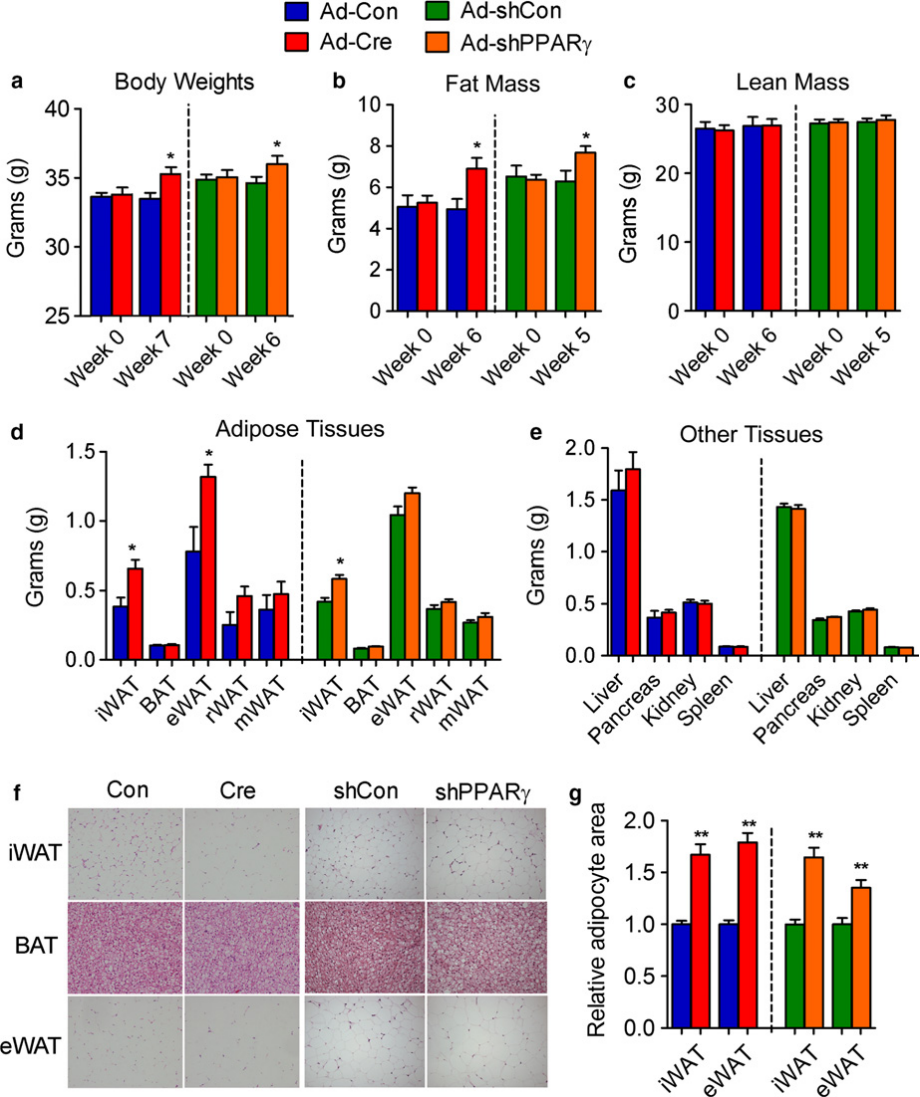
PPARγ deficiency in iWAT of aging mice increases adiposity. (a–g) Analysis of 12‐month‐old control, PPARγ‐iWAT‐KO, and PPARγ‐iWAT‐KD mice. *n *= 6 per group. (a) Body weight; (b) fat mass; (c) lean mass; (d) adipose tissue weights; (e) tissue weights; (f) representative images of H&E staining of adipose tissues; (g) adipocyte sizes of iWAT and eWAT. Data are presented as mean ± SEM and *, *p* < .05; **, *p* < .01

We next assessed the effects of PPARγ modulation specifically in subcutaneous tissues. Mid‐aged PPARγ‐iWAT‐KO and PPARγ‐iWAT‐KD mice showed increased total body weight (Figure 2a), and nuclear magnetic resonance (NMR) scan assessment of body composition revealed a selective increase in fat mass while no changes in lean mass were observed (Figure 2b,c). Detailed measurements of adipose depots revealed an increase in the amounts of fat tissues (Figure 2d), while the weight of organs such as pancreas, kidney, and spleen was not altered (Figure 2e). Further histological analysis showed increased lipid accumulation in all three fat depots, as revealed by H and E staining (Figure 2f) and increased adipocyte size (Figure 2g) compared to controls.

### 2.3 Impaired glucose and lipid metabolism in mice with PPARγ deficiency in iWAT

**Figure 3:**
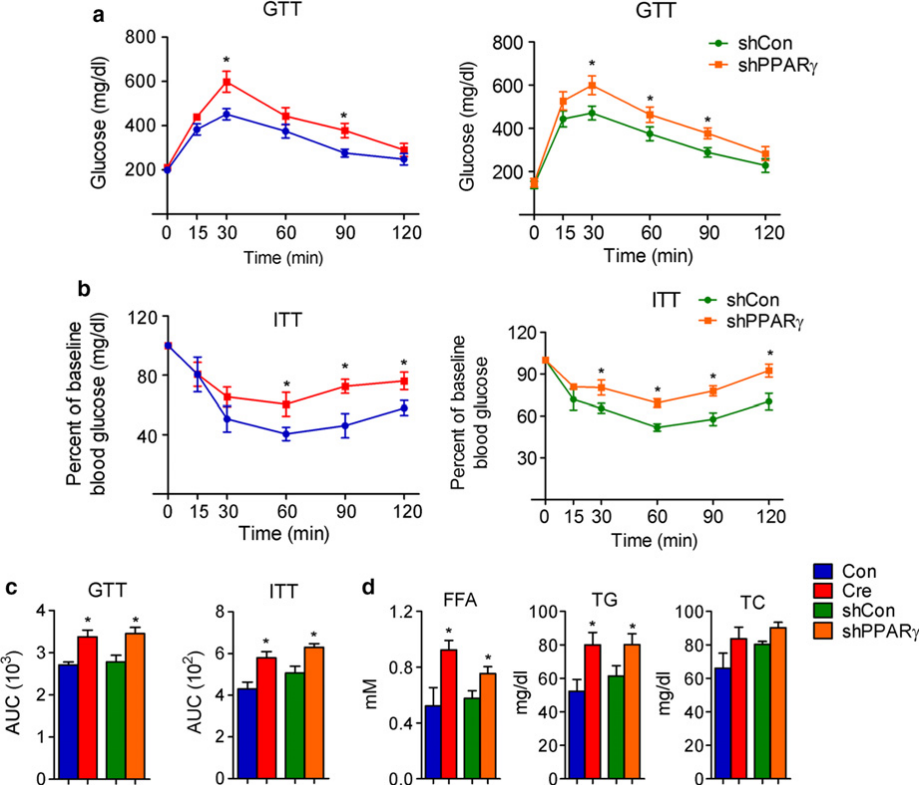
Impaired glucose and lipid homeostasis in aging mice with PPARγ deficiency in iWAT. (a–d) Analysis of 12‐month‐old control, PPARγ‐iWAT‐KO, and PPARγ‐iWAT‐KD mice. *n *= 6 per group. (a, b, c) Insulin sensitivity measured by GTT and ITT and shown as area under the curve (AUC); (d) serum parameters including free fatty acids (FFA), total triglyceride (TG), and total cholesterol (TC). Data are presented as mean ± SEM and *, *p* < .05; **, *p* < .01

We next examined the impact of PPARγ deficiency on glucose and lipid homeostasis in mid‐aged mice with modulation of PPARγ levels specifically in iWAT. Analysis of metabolic parameters revealed impaired glucose and insulin tolerance (Figure 3a–c). Furthermore, mice with PPARγ deficiency in iWAT showed significantly increased serum‐free fatty acid and triglycerides and a trend of higher cholesterol levels (Figure 3d). These results indicate that aging mice with PPARγ deficiency in iWAT are more insulin resistant than control mice and have abnormal lipid profiles.

### 2.4 Decreased energy expenditure and brown fat gene expression in mice with PPARγ deficiency in iWAT

**Figure 4:**
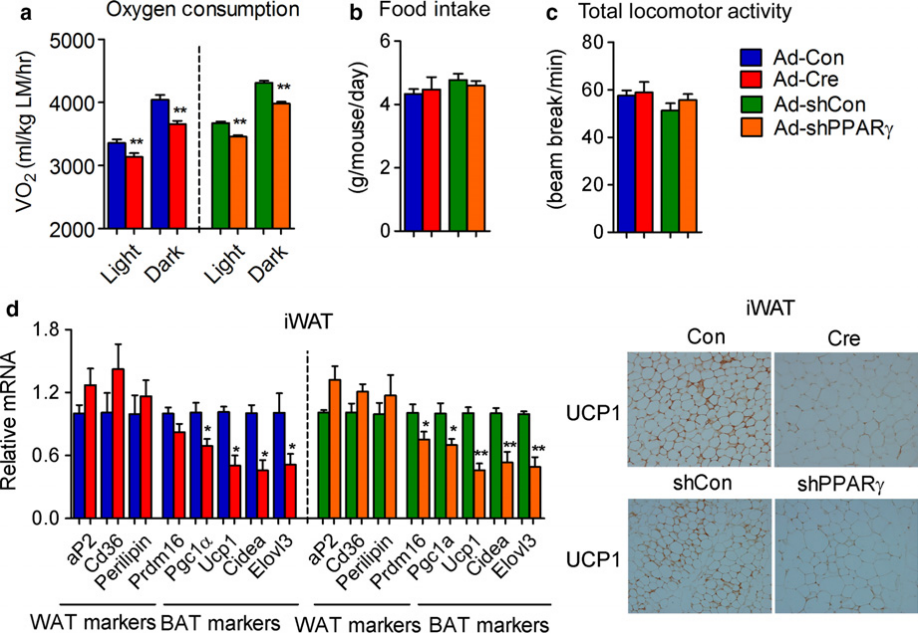
PPARγ is required for the maintenance of brown fat programs in iWAT of aging mice. (a–e) Analysis of 12‐month‐old control, PPARγ‐iWAT‐KO, and PPARγ‐iWAT‐KD mice. *n *= 6 per group. (a) Oxygen consumption normalized to lean mass (LM); (b) food intake; (c) total locomotor activity; (d) gene expression levels of white and brown markers in iWAT; (e) representative images of UCP1 staining in iWAT. Data are presented as mean ± SEM and *, *p* < .05; **, *p* < .01

We next compared the metabolic performance of control mice with that of mice with PPARγ deficiency in iWAT. As shown in Figure 4a, PPARγ‐iWAT‐KO and PPARγ‐iWAT‐KD showed decreased whole‐body oxygen consumption. CLAMS analysis revealed that changes in oxygen consumption were not accompanied by altered food intake nor of locomotor activity (Figure 4b,c). Detailed molecular analysis demonstrated preferential impairment of brown fat gene programs, with no changes in white gene programs (Figure 4d), and immunohistochemistry of iWAT revealed decreased UCP1 staining in mice with PPARγ deficiency (Figure 4e). Together, these data indicate that PPARγ is required for the maintenance of brown gene programs in iWAT during aging.

### 2.5 Age‐dependent differential control of white and brown gene programs in inguinal fat by PPARγ

**Figure 5:**
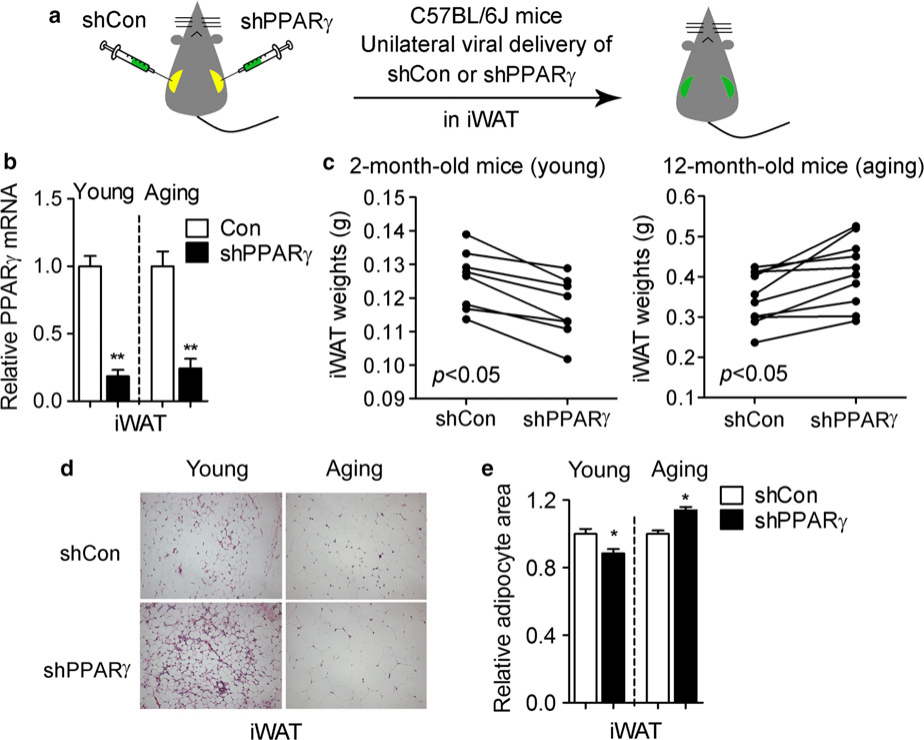
PPARγ deficiency in iWAT of young and aging mice leads to distinct adiposity phenotypes. (a) Illustration of the strategy used to reduce PPARγ levels in the iWAT of young and aging mice via adenoviral delivery. (b) PPARγ mRNA levels; (c) weight of iWAT; (d) representative H&E images of iWAT and (e) adipocyte area in 2‐month‐old and 12‐month‐old mice injected with shRNA control (shCon) or shPPARγ (shPPARγ) adenoviruses. Data are presented as mean ± SEM and *, *p* < .05; **, *p* < .01

To further examine whether PPARγ controls gene programs in an age‐ and depot‐dependent manner, we downregulated PPARγ levels in iWAT of young and aging mice via unilateral injections in subcutaneous fat of control or shPPARγ adenoviruses (Figure 5a,b). Phenotypical and molecular analysis revealed that PPARγ knockdown in iWAT of young mice is associated with decreased fat amount and reduced adipocyte size (Figure 5c–e), consistent with the lipodystrophic phenotype previously reported in fat‐specific PPARγ knockout mice (He et al., 2003; Jones et al., 2005; Wang et al., 2013). In contrast, decreasing levels of PPARγ selectively in iWAT in aging mice led to an increase in both the amount of subcutaneous fat and in the size of its adipocytes (Figure 5c–e).

**Figure 6:**
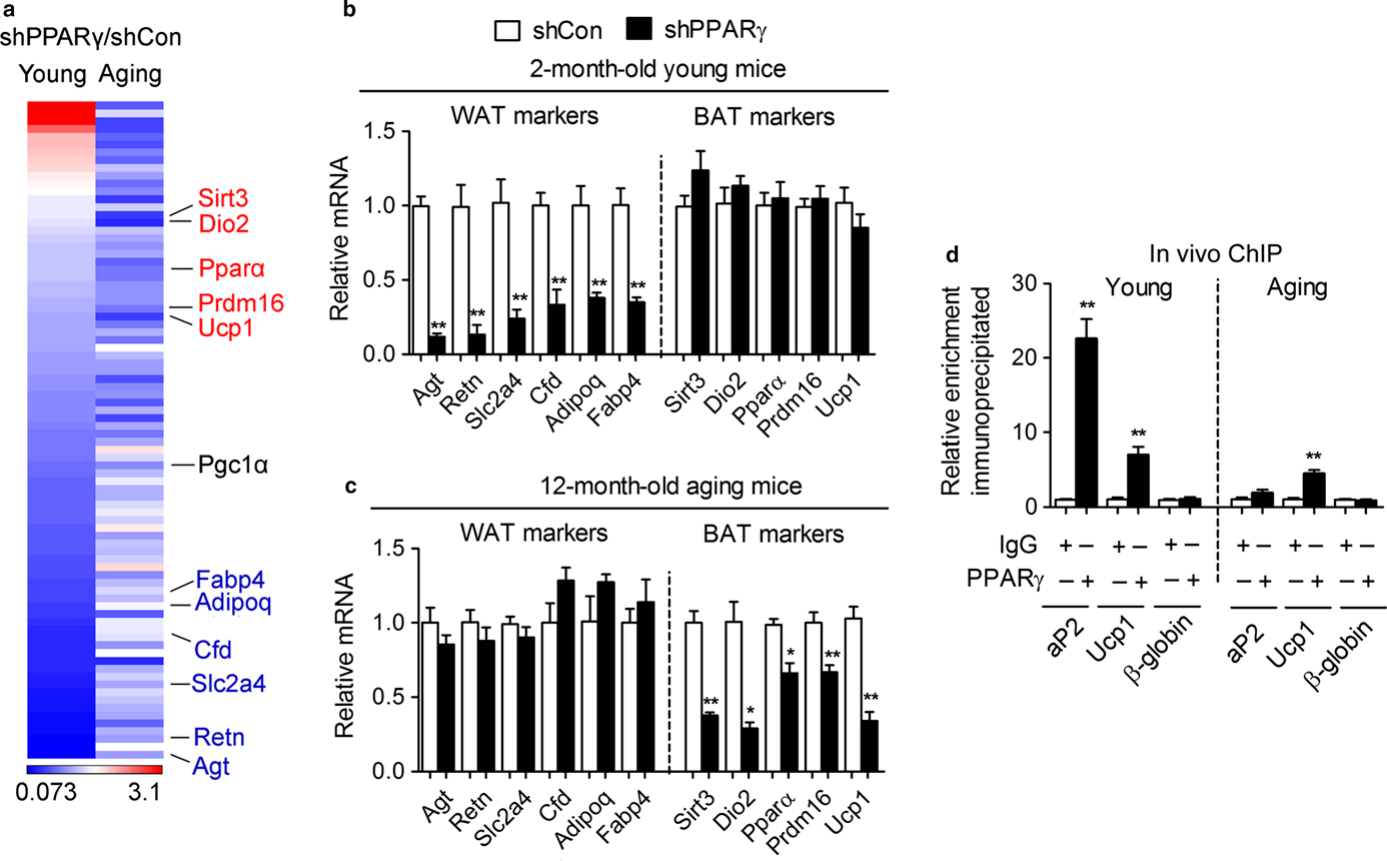
PPARγ preferentially regulates brown gene programs in inguinal fat of aging mice. (a) Heat map of white and brown gene transcripts in iWAT of 2‐month‐old (young) and 12‐month‐old (aging) mice with knockdown of PPARγ (shPPARγ) compared to control (shCon) (b, c) White and brown PPARγ gene targets in iWAT of 2‐month‐old (b) and 12‐month‐old (c) mice after PPARγ knockdown. (d) Chromatin IP at the aP2 promoter and at the Ucp1 enhancer in iWAT of 2‐month‐old (Young) and 12‐month‐old (Aging) mice. The β‐globin promoter was used as a negative control. Data are presented as mean ± SEM and *, *p *<* *.05; **, *p *<* *.01. *n *= 7–8 per group

Unbiased gene array analyses of iWAT obtained from young and old mice injected with control or shPPARγ revealed that young mice with PPARγ knockdown have a selective reduction in the expression of white fat gene targets such as Agt, Retn/Resistin, Slc2a4/Glut4, Cfd/Adiposin, Adipoq/Adiponectin, and Fabp4/aP2 (Figure 6a,b), while downregulation of PPARγ in aging mice affects specifically brown fat genes such as Dio2, Pparα, Prdm16, and Ucp1 (Figure 6a,c). Of note, the levels of a known PPARγ target gene, Pgc1α, appeared to be regulated by PPARγ deficiency in both young and aging mice (Table S1 and Figure 6a), further demonstrating that PPARγ downregulation affects only select gene subsets. Interestingly, PPARγ suppression only affected expression levels of genes for lipid accumulation in young mice but not in aging mice (Fig. S2), suggesting that other factors may be able to complement PPARγ function on lipid accumulation during aging. To assess whether changes in PPARγ levels occur during the aging process, we measured PPARγ mRNA and protein in iWAT of young and aging mice. Although we did not observe any difference in the total amount of PPARγ mRNA and protein, our analysis revealed higher levels of PPARγ phosphorylation at Serine 273 in aging compared to young mice (Fig. S3). Next, we assessed possible changes in the levels of occupancy of PPARγ at the promoters of aP2 and Ucp1 occurring in aging, given that those genes represent markers of white and brown fat programs, respectively. As shown in Figure 6d, while PPARγ is predominantly present at the PPRE site of the aP2 promoter in subcutaneous tissues obtained from young mice, in aging mice PPARγ appears preferentially bound to the Ucp1 enhancer. Collectively, these data suggest that PPARγ deficiency in iWAT may differentially affect white and brown fat gene programs depending on the age of the mice.

## 3 DISCUSSION

It has long been noted that during aging, obesity and metabolic dysfunction often ensues; however, to date, the transcriptional switches turned on during the aging process and responsible for the metabolic changes observed late in life have not yet been fully characterized. Given the importance of PPARγ in fat tissue biology, in this study we sought to determine the role of PPARγ in aging‐associated metabolic decline. Through the use of two adenoviral‐based in vivo methodologies, we have provided for the first time evidence to support a novel and critical requirement of PPARγ for the maintenance of browning programs in subcutaneous tissue during aging. Given the recent demonstration that beige fat cells interspersed in inguinal fat tissue expend energy via creatine metabolism (Kazak et al., 2015), it is of interest to assess whether the effects of PPARγ reported here involve alternative futile cycles in addition to classical thermogenic pathways.

The results of our studies showing that PPARγ deficiency selectively in subcutaneous fat during aging is associated with increased adiposity are surprising given that they are sharply in contrast with the lipodystrophic phenotype and the impairment in adipose tissue expansion previously reported in aP2‐ and adiponectin‐driven fat‐specific PPARγ KO mice (He et al., 2003; Jones et al., 2005; Wang et al., 2013) and in young mice with decreased PPARγ levels selectively in iWAT (Figure 5). The striking dissimilarity between the effects of PPARγ ablation on fat tissue reported in published aP2‐ and adiponectin‐driven knockout models and in our study of young mice may be due to the differences in the spatiotemporal conditions of PPARγ ablation, given that PPARγ deletion was previously achieved in every fat depot during development and in adult mice (He et al., 2003; Jones et al., 2005; Wang et al., 2013), while here PPARγ levels are selectively reduced in subcutaneous fat tissue in mid‐aged mice. It is conceivable that the animal model tested here may have allowed the specification of the select fat depot and life stage in which one of the two opposed PPARγ functions, adipogenic and thermogenic, is predominant. Given that it has been recently shown that PPARγ gene target selection is dictated by depot‐selective coregulators, such as TLE3 and Prdm16, which can specify alternative programs of lipid storage or thermogenesis (Koppen & Kalkhoven, 2010; Peirce, Carobbio & Vidal‐Puig, 2014; Villanueva et al., 2013), it can be envisioned that preponderance of one type of cofactor in an aging tissue may drive PPARγ to activate only select gene targets. Future studies will determine whether the preferential binding of PPARγ to brown fat gene promoters we observed in 12‐month‐old mice is driven by differential amounts of brown versus white cofactors present in subcutaneous adipose tissues in aging mice.

It is also plausible that posttranslational modifications in PPARγ occurring specifically in aging could modify PPARγ target gene promoter binding choices by potentially altering PPARγ affinity for specific cofactors, given that it has been previously demonstrated that the recruitment of brown fat coactivators can be modulated by the PPARγ acetylation status in young mice (Qiang et al., 2012) and that phosphorylation of PPARγ promotes the interaction with specific coregulators (Choi et al., 2014). Here, we show for the first time that the phospho‐status of PPARγ is modified during the aging process; whether phosphorylation at serine 273 in PPARγ may direct the selective expression of target genes in an age‐specific manner remains to be determined.

Our results provide the first evidence that, during aging, PPARγ in subcutaneous adipose tissue may be important in the control of energy expenditure. Furthermore, our data highlight possible differential metabolic effects of PPARγ activity at different life stages. In particular, our comparison of the effects of ablation of PPARγ in young and aging mice suggests that PPARγ's role in mature adipocytes shifts during aging from the maintenance of lipogenic functions to the control of energy expenditure and suggest that other transcription factors may complement PPARγ function in lipid accumulation during aging. Overall, the present study offers a broader view of the function of PPARγ during aging than previously appreciated and provides a new rationale for targeting PPARγ to increase energy expenditure at late life stages and to reduce, or prevent, age‐associated metabolic decline.

## 4 EXPERIMENTAL PROCEDURES

### 4.1 Animal studies

All mouse studies were performed according to guidelines of the National Institute of Diabetes and Digestive and Kidney Diseases Animal Care and the Animal Ethics Committee of East China Normal University. Mice were housed at room temperature in 12‐hr light/dark cycles with ad libitum access to a standard chow diet (NIH‐31; Harlan Laboratories, Bethesda, MD, USA) and water. Body composition was assessed via an Echo NMR analyzer (Echo Medical Systems, Houston, TX, USA). Oxygen consumption, food intake, and locomotor activity were measured with CLAMS (Columbus Instruments, Columbus, OH, USA) at 22oC after two days of adaptation.

### 4.2 Adenoviral delivery into inguinal fat

PPARγ flox/flox mice were purchased from Jax laboratory. Adenoviruses expressing control (CMV‐GFP), Cre (Cre‐CMV‐GFP), control shRNA (U6‐shRNA‐CMV‐GFP), and shPPARγ (U6‐shPPARγ‐CMV‐GFP) were constructed, amplified, and purified by Vector BioLabs, Malvern, PA. 50 μl of each adenovirus diluted in saline was injected unilaterally (5 × 10^9^ pfu, for acute purposes) or bilaterally (2 × 10^9^ pfu, for chronic purposes) into the inguinal fat pads of mice (Ma, Xu, Gavrilova & Mueller, 2014; Ma et al., 2015; Xu, Ma, Bagattin & Mueller, 2016). For acute analysis, mice were euthanized on the fourth day after viral delivery and for long‐term studies, mice were injected once a week for up to 6 weeks.

### 4.3 Isolation of adipose macrophage (ATM) from inguinal fat

Inguinal fat from mice was excised under sterile conditions and fractionated to obtain stromal vascular cells (SVF), as previously described (Ma et al., 2015). Briefly, inguinal fat was minced and subjected to collagenase (1 mg/ml) digestion at 37°C for 45 min in buffer containing 0.123M NaCl, 5 mm KCl, 1.3 mm CaCl2, 5 mm glucose, 100 mm Hepes, and 4% BSA, filtered through a 100‐um nylon screen and centrifuged at 150 g for 5 min at room temperature. F4/80‐positive ATMs were selected from the total SVF using the MACS Microbeads technology (Miltenyi Biotec, Bergisch Gladbach, Germany) according to the manufacturer's instructions. Briefly, the SVFs were magnetically labeled by incubating them with mouse F4/80‐biotin antibody (Miltenyi Biotec, 130‐101‐893) and antibiotin microbeads (Miltenyi Biotec, 130‐090‐485) and passed through the MS separation column (Miltenyi Biotec, 130‐042‐201) while placed in the magnetic field of a MidiMACS separator (Miltenyi Biotec). F4/80‐positive cells were removed from the column with 2 ml of MACS buffer, twice, and stored for further analysis.

### 4.4 Serum analysis and insulin sensitivity

Serum triglyceride (Thermo, Waltham, MA, USA), total cholesterol (Sigma, St. Louis, MO) and free fatty acid (Roche, Indianapolis, IN, USA) levels were assayed by colorimetric tests. For insulin and glucose tolerance tests, mice received an intraperitoneal injection of insulin (1 mU/kg; Humulin, Lilly, Indianapolis, IN, USA) in random‐fed state or a glucose solution in saline (1.5 g/kg) after an overnight fast. Plasma glucose levels were measured from tail blood before or 15, 30, 60, 90, and 120 min after insulin or glucose injections via automatic reader (Bayer, Leverkusen, Germany). AUC (area under the curve) was calculated with GraphPad software, as previously described (Ma, Xu & Mueller, 2016).

### 4.5 Histological analysis

Dissected tissues were fixed in 10% neutral‐buffered formalin and embedded in paraffin according to standard procedures. Tissue sections of 5 μm thickness were stained with hematoxylin and eosin (Histoserv, Germantown, MD, USA) or with antibodies against UCP1 (ab10983), GFP (ab290), and F4/80 (Ab6640) from Abcam, Cambridge, MA, USA, following the manufacturer's instructions (Vector Laboratories, Burlingame, CA, USA). Quantification of adipocyte size was performed on ImageJ, and 10 random fields were selected from each slide. Quantification of immunostaining of GFP and F4/80 was performed with 15 random fields per slide obtained from five mice by confocal microscopy (Leica, SP8, Germany).

### 4.6 Real‐time PCR and PCR array

Total RNA was extracted from tissues with TRIzol (Invitrogen, Waltham, MA, USA) or RNeasy (Qiagen), and 1 μg total RNA was reverse‐transcribed to cDNA with First Strand cDNA Synthesis Kit (Roche). Quantitative real‐time PCR was performed with the ABI PRISM 7900HT sequence detection system (Applied Biosystems, Waltham, MA, USA) using SYBR green (Roche). Gene expression levels were determined by the delta‐delta Ct method, after normalization to 36B4 expression. Primer sequences are listed in Table S2. PCR array was purchased from Qiagen (RT² Profiler™ PCR Array Mouse Adipogenesis, PAMM049Z, Valencia, CA, USA) and used according to manufacturer's protocol.

### 4.7 Western blot

Protein extraction was performed with RIPA buffer consisting of 20 mm Tris, 150 mm NaCl, 1% Triton X‐100, and protease inhibitors (Roche) and loaded on a 10% Bis‐Tris Gel and transferred to PVDF membranes (Pierce, Waltham, MA, USA) and incubated with anti‐PPARγ (Santa Cruz, sc‐7196, CA, USA), anti‐PPARγ phospho 273 (Bioss, 4888R, MA, USA), and anti‐β‐actin (Sigma, A5316, St. Louis, MO, USA) antibodies.

### 4.8 In vivo chromatin immunoprecipitation assays

For in vivo ChIP analysis, inguinal fat was first processed as previously reported (Haim, Tarnovscki, Bashari & Rudich, 2013). Briefly, inguinal fat tissues were freshly dissected, minced in small pieces, and cross‐linked with 1.5% formaldehyde and subsequently treated with 0.125 m glycine. After incubation, samples were centrifuged at room temperature at 2,500 rpm for 5 min and placed on ice. The upper phase including lipid‐rich tissue pieces and fat was washed twice with ice‐cold PBS supplemented with protease inhibitors (Roche) followed by centrifugation (5 min, 2,500 rpm, 4°C). After removal of the liquid phase, small adipose tissue pieces were resuspended in adipocyte lysis buffer containing 500 mm PIPES, 80 mm KCl, and 1% Igepal (Sigma) supplemented with protease inhibitors, homogenized using a Dounce homogenizer (Thomas Scientific, Swedesboro, NJ, USA) and incubated on ice for 15 min by vortexing. Larger particles were removed using a 250‐μm mesh. Then, samples were centrifuged (5 min, 2,500 rpm, 4°C) and the pellet of nuclei was resuspended in 500 μl of SDS lysis buffer supplemented with protease inhibitors and incubated on ice for 20 min prior to sonication. The following steps were performed according to the standard protocols described in the manuals accompanying the ChIP assay kit (Millipore, Billerica, MA, USA). The primer sequences were the following: to assess binding at the Ucp1 enhancer: Forward: TGAGGCTGATATCCCCAGAGA, Reverse: TCTGTGTGTCCTCTGGGCATAA; to detect occupancy at the aP2 promoter Forward: TTCCCAGCAGGAATCAGGTAG, Reverse: CTGGGAACTCCATTTGCTCTC; to detect binding at the β‐globin promoter Forward: AAGCCTGATTCCGTAGAGCCACAC, Reverse: CCCACAGGCA AGAGACAGCAGC.

### 4.9 Statistical analysis

Student's *t* test was used for comparison between two groups using GraphPad software. Paired *t* test was used to compare iWAT weights with unilateral adenoviral delivery of control or shPPARγ in iWAT of 2‐ and 12‐month‐old mice by SPSS software. *p *<* *.05 was considered as statistically significant. Results are shown as mean ± SEM.

## AUTHOR CONTRIBUTIONS

L.X and X.M devised, designed, and performed experiments and analyzed results. N.K.V. and D.M.W performed experiments and analyzed results. O.G. measured energy expenditure and serum parameters. R.L.P. and T.F. participated in the experimental design and in the interpretation of the results. X.M., L.X., and E.M. wrote the manuscript. E.M. conceived the project and coordinated its execution. All authors commented and approved the manuscript.

## CONFLICT OF INTEREST

The authors have declared no conflict of interest
