# Supplementary figures and images for: Ablation of PPARγ in subcutaneous fat exacerbates age‐associated obesity and metabolic decline
Source: Aging Cell. 2018 Jan 31;17(2):e12721. doi: 10.1111/acel.12721 (PMC5847881; doi:10.1111/acel.12721)

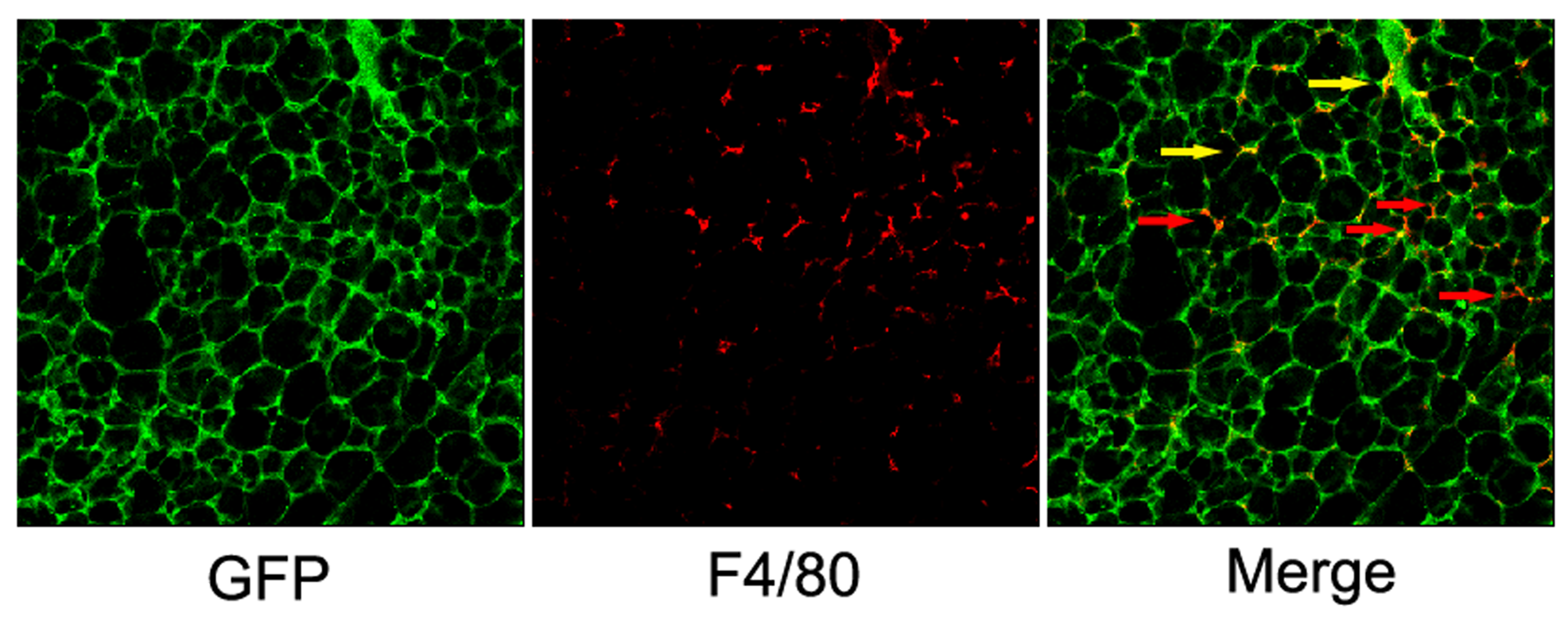

Supplement: Supplementary file 1 [file ACEL-17-e12721-s001.tif]

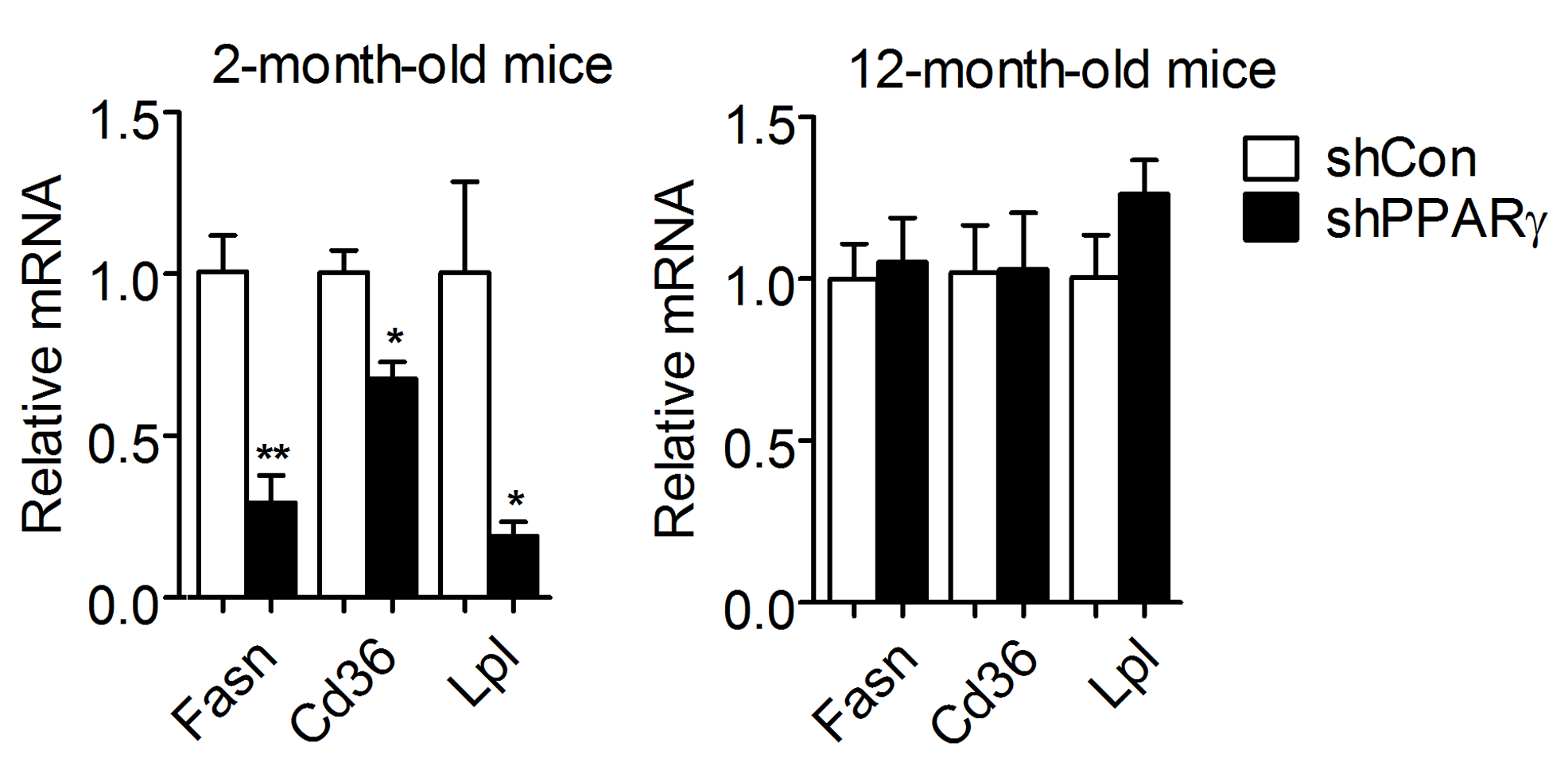

Supplement: Supplementary file 2 [file ACEL-17-e12721-s002.tif]

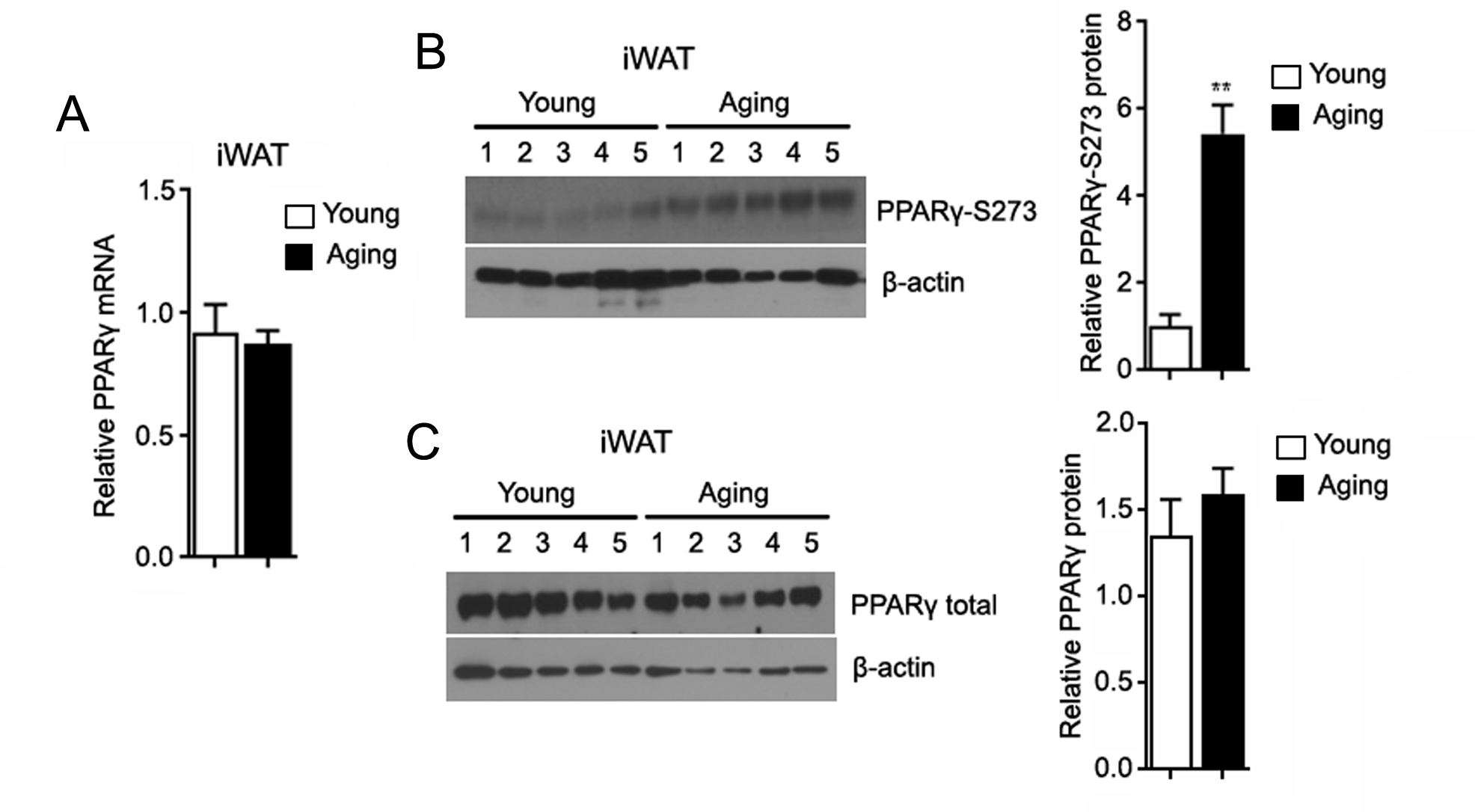

Supplement: Supplementary file 3 [file ACEL-17-e12721-s003.tif]
