# Supplementary material for: Ablation of PPARγ in subcutaneous fat exacerbates age‐associated obesity and metabolic decline
Source: Aging Cell. 2018 Jan 31;17(2):e12721. doi: 10.1111/acel.12721 (PMC5847881; doi:10.1111/acel.12721)
Supplement: Supplementary file 4 [file ACEL-17-e12721-s004.pdf]

Supplemental Table S1. Adipogenesis PCR-array results.

Gene	Young	Aging	Gene	Young	Aging
Wnt1	3.08**	0.52**	Gata3	1.05	0.54**
Nrob2	3.07**	1.12	Ppara	1.02	0.67*
Vdr	2.52**	0.42**	Foxc2	1.01	0.65*
Wnt3a	2.09**	0.45**	Ppargc1b	1.01	0.81
Dkk1	1.64*	0.58*	Runx1t1	0.98	0.81
Dlk1	1.63*	0.48*	Bmp4	0.95	0.81
Src	1.58*	0.69*	Prdm16	0.93	0.67*
E2f1	1.55	0.57*	Ucp1	0.91	0.39**
Ccnd1	1.50	1.05	Axin1	0.91	0.65*
Shh	1.42	0.85	Adrb2	0.91	0.93
Bmp2	1.34	0.60*	Fgf1	0.90	0.67*
Egr2	1.32	0.76	Cdkn1a	0.89	1.30
Wnt5b	1.22	0.47**	Wnt5a	0.86	1.01
Bmp7	1.23	1.09	Acacb	0.86	0.87
Sirt3	1.22	0.38**	Mapk14	0.85	0.86
Dio2	1.17	0.29**	Creb1	0.79	0.49**
Wnt10b	1.14	1.04	Hes1	0.79	0.76
Angpt2	1.08	0.89	Cebpd	0.78	0.79
Nrf1	1.05	0.78	Sfrp1	0.78	0.51**
Ncoa2	1.05	0.91	Ppard	0.75	0.92

Gene	Young	Aging	Gene	Young	Aging
Gata2	0.73	0.43**	Jun	0.45**	0.75
Cebpb	0.72	0.90	Ncor2	0.44**	1.01
Klf2	0.68*	0.66*	Fabp4	0.41**	1.12
Nr1h3	0.65*	0.91	Sirt2	0.41**	0.97
Lrp5	0.67*	1.44	Adipoq	0.38**	1.26
Cdkn1b	0.64*	1.13	Sirt1	0.36**	0.53**
Ppargc1a	0.63*	0.72*	Adig	0.32**	1.23
Rxra	0.61**	1.01	Cfd	0.30**	1.21
Cdk4	0.58*	1.21	Cebpa	0.30**	1.18
Ddit3	0.57**	0.95	Klf15	0.28**	0.77
Lmna	0.56*	0.92	Lpl	0.28**	1.30
Tcf7l2	0.56*	1.12	Pparg	0.28**	0.27**
Klf4	0.56**	1.21	Klf3	0.27**	0.95
Rb1	0.54**	1.07	Fasn	0.26**	1.09
Twist1	0.54**	1.42	Slc2a4	0.23**	0.91
Fgf2	0.54**	0.84	Srebf1	0.21**	1.13
Irs2	0.50**	1.03	Irs1	0.19**	1.03
Insr	0.49**	0.98	Lep	0.17**	0.95
Foxo1	0.49**	1.09	Tsc22d3	0.15**	0.87
Taz	0.48**	1.40	Sfrp5	0.13**	0.56*

Gene	Young	Aging
Lipe	0.13**	0.92
Retn	0.10**	0.84
Fgf10	0.08**	1.34
Agt	0.07**	0.84

PCR-arrays (Qiagen, PAMM049Z) were used to determine fold change of relative gene expressions in subcutaneous fat with adenoviral delivery of shPPARγ vs shCon in young (2-month-old) and aging (12-month-old) mice. *, $P<0.05$; **, $P<0.01$ and n=3 per group.
