# Supplementary material for: Ablation of PPARγ in subcutaneous fat exacerbates age‐associated obesity and metabolic decline
Source: Aging Cell. 2018 Jan 31;17(2):e12721. doi: 10.1111/acel.12721 (PMC5847881; doi:10.1111/acel.12721)
Supplement: Supplementary file 5 [file ACEL-17-e12721-s005.pdf]

Supplemental Table S2. The sequences of primers used in Real-Time PCR.

Primer	Forward Primer 5'-3'	Reverse Primer 5'-3'
m36B4	GCTTCATTGTGGGAGCAGAC	ATGGTGTTCTTGCCCATCAG
mPgc1α	ACCATGACTACTGTCAGTCACTC	GTCACAGGAGGCATCTTTGAAG
mUcp1	GGCCCTTGTAAACAACAAAATAC	GGCAACAAGAGCTGACAGTAAAT
mPrdm16	CCACCAGCGAGGACTTCAC	GGAGGACTCTCGTAGCTCGAA
mCidea	TGACATTCATGGGATTGCAGAC	CGAGCTGGATGTATGAGGGG
mElovl3	TTCTCACGCGGGTTAAAAATGG	TCTCGAAGTCATAGGGTTGCAT
maP2	ACACCGAGATTTCCTTCAAACTG	CCATCTAGGGTTATGATGCTCTTC
mCd36	TTTGGAGTGGTAGTAAAAAGGGC	TGACATCAGGGACTCAGAGTAG
mPerilipin	CTGTGTGCAATGCCTATGAGA	CTGGAGGGTATTGAAGAGCCG
mPparγ	TCCAGCATTTCTGCTCCACA	ACAGACTCGGCACTCAATGG
mAgt	TCTCCTTTACCACAACAAGAGCA	CTTCTCATTCACAGGGGAGGT

mRetn	ACAAGACTTCAACTCCCTGTTTC	TTTCTTCACGAATGTCCCACG
mSlc2a4	GTGACTGGAACACTGGTCCTA	CCAGCCACGTTGCATTGTAG
mCfd	CATGCTCGGCCCTACATGG	CACAGAGTCGTCATCCGTCAC
mAdipoq	TGTTCCTCTTAATCCTGCCCA	CCAACCTGCACAAGTTCCCTT
mSirt3	ATCCCGGACTTCAGATCCCC	CAACATGAAAAAGGGCTTGGG
mDio2	AATTATGCCTCGGAGAAGACCG	GGCAGTTGCCTAGTGAAAGGT
mPparα	AGAGCCCCATCTGTCCTCTC	ACTGGTAGTCTGCAAAACCAAA
mFasn	GGAGGTGGTGATAGCCGGTAT	TGGGTAATCCATAGAGCCCAG
mLpl	GGGAGTTTGGCTCCAGAGTTT	TGTGTCTTCAGGGGTCCTTAG
